# Multiplexed optoacoustic tracking and magnetic actuation of labeled blood cells in living mice

**DOI:** 10.1126/sciadv.aec8985

**Published:** 2026-06-26

**Authors:** Lin Tang, Quanyu Zhou, Hanna Preuss, Xuyang Chang, Lukas Glandorf, Daniil Nozdriukhin, Etienne Jessen, Chaim Glück, Hequn Zhang, Yi Chen, Michael Reiss, Zhe Feng, Dominik Schillinger, Bruno Weber, Susanne Wegener, Mohamad El Amki, Daniel Razansky, Xosé Luís Deán-Ben

**Affiliations:** ^1^ Institute of Pharmacology and Toxicology, University of Zurich, 8057 Zurich, Switzerland.; ^2^ Institute for Biomedical Engineering, Department of Information Technology and Electrical Engineering, ETH Zurich and University of Zurich, 8093 Zurich, Switzerland.; ^3^ Department of Neurology, University Hospital and University of Zurich, 8091 Zurich, Switzerland.; ^4^ Neuroscience Center Zurich, University of Zurich and ETH Zurich, 8057 Zurich, Switzerland.; ^5^ Institute for Mechanics, Computational Mechanics Group, Technical University of Darmstadt, 64287 Darmstadt, Germany.; ^6^ Eye Research Center, Hangzhou Institute of Medicine, Chinese Academy of Sciences, Eye Hospital, Wenzhou Medical University, 310018 Hangzhou, China.; ^7^ State Key Laboratory of Extreme Photonics and Instrumentations, Centre for Optical and Electromagnetic Research, College of Optical Science and Engineering, International Research Center for Advanced Photonics, Zhejiang University, 310058 Hangzhou, China.

## Abstract

Visualization of cellular dynamics in microvascular networks is essential for deciphering physiology and disease. Existing imaging platforms commonly lack the spatiotemporal resolution and sensitivity for single-cell tracking in vivo, particularly beyond the penetration depth of optical microscopy. While recent fluorescence and optoacoustic methods allow detection of single microparticles in vivo, multiplexed imaging of distinct circulating cell populations remains unachieved. Here, we present an optimized labeling protocol using near-infrared (NIR) dyes ICG (indocyanine green) and DiR (1,1′-dioctadecyl-3,3,3′,3′-tetramethylindotricarbocyanine iodide) to generate spectrally distinct optoacoustic signatures for red blood cells (RBCs) and neutrophils. This enables noninvasive simultaneous tracking of both cell types in deep cerebrovascular networks of mice, supporting superresolution imaging with localization optoacoustic tomography and revealing differences in the velocities of different cell types inside opaque tissues. ICG-labeled RBCs are further detectable via diffuse optical localization imaging in the NIR-II window, permitting high-resolution visualization of cortical capillaries. In addition, incorporation of superparamagnetic nanoparticles allows noninvasive magnetic manipulation of the microcirculation alongside real-time single-cell monitoring, further providing a strategy for precise and reversible vascular occlusion in preclinical ischemic stroke models. Together, these capabilities provide a versatile platform for advancing vascular research at the single-cell level.

## Teaser

Engineered blood cells enable in vivo tracking, discrimination, and magnetic actuation.

## INTRODUCTION

Microvascular networks comprising arterioles, venules, and capillaries constitute a highly regulated system in which different types of cells coordinate oxygen delivery, immune surveillance, and waste removal through intricate hemodynamic processes (*1*–*3*). Red blood cells (RBCs) undergo deformation to maintain capillary flow and oxygen delivery (*4*), while neutrophils and other leukocytes precisely control their adhesion and transmigration to mediate immune surveillance (*5*). These distinct cellular dynamics are critical for maintaining tissue homeostasis and play a pivotal role in various pathological conditions (*6*, *7*).

Real-time monitoring and tracking of cellular dynamics have been pivotal for understanding physiological processes and disease mechanisms (*8*). Optical microscopy, including bright-field, phase contrast, and fluorescence (FL)–based techniques, has served as a critical tool for visualizing cell migration, proliferation, and other key behaviors in cultured cells (*9*–*11*). In vivo optical methods, particularly based on FL contrast, have further enabled visualization of individual circulating cells, providing essential insights into hemodynamics and cellular interactions (*12*–*14*). However, optical imaging is inherently limited by light scattering, restricting high-resolution visualization to superficial depths of a few hundred micrometers (*15*), which is generally insufficient to resolve subcutaneous microvascular networks in mammalian tissues in a noninvasive fashion. Labeling of cells for deep tissue imaging has been achieved, e.g., with radiotracers in single-photon emission computed tomography (SPECT) (*16*) or positron emission tomography (PET) (*17*), superparamagnetic iron oxide nanoparticles (SPIONs) in magnetic resonance imaging (MRI) (*18*, *19*), and more recently, gas vesicles in ultrasound (*20*, *21*). However, these methods remain limited by insufficient spatiotemporal resolution for tracking fast cellular dynamics and lack multiplexing capability for distinguishing specific cell populations.

The versatility of optical contrast mechanisms has driven the development of optoacoustic (OA; photoacoustic) imaging as a solution to overcome the fundamental depth limitations of optical microscopy in living mammalian tissues [~1 mm at near-infrared (NIR) wavelengths] (*22*–*25*). OA microscopy achieves a single-cell resolution through optical focusing at the tissue surface, thus enabling label-free cell tracking in superficial tissue layers (*25*). By contrast, OA tomography offers a larger field of view (FOV) and deeper tissue penetration but is uncapable of achieving a capillary-level resolution (*24*). The high-frame-rate capacity of state-of-the-art OA tomography has enabled tracking of individual circulating melanoma tumor cells (*26*, *27*). Therefore, promising avenues have emerged to uncover previously inaccessible spatiotemporal patterns of vascular trafficking. Strong absorbers loaded with high-extinction-coefficient dyes, such as IR780 droplets, have enabled single-particle detection and localization amid the strong blood background absorption, paving the way for the recent development of localization OA tomography (LOT) (*28*–*30*). This superresolution modality efficiently synergizes three-dimensional (3D) microangiography and blood flow mapping with unique OA functional and molecular imaging capabilities (*29*, *31*, *32*). It has further been demonstrated that some of these particles can be magnetically steered, opening new possibilities for targeted drug delivery, localized hyperthermia therapy, tissue engineering, or diagnostic sensing (*33*, *34*). However, LOT has faced two critical limitations, namely (i) biocompatibility risks, including potential vascular occlusion, associated with its dependence on extrinsically administered microparticles, and (ii) lack of efficiency and specificity of the existing labeling strategies for reliable single-cell tracking and phenotypic identification in physiologically relevant environments.

The so-called second NIR (NIR-II) spectral window (900 to 1700 nm) represents a promising alternative for deep-tissue optical imaging beyond the conventional depth limitations of FL microscopy. The recent development of sensitive short-wave infrared (SWIR) cameras operating in this spectral range opened a window characterized by reduced scattering and autofluorescence, thus allowing for deeper penetration of ballistic photons. This has been exploited in diffuse optical localization imaging (DOLI) using NIR-II–emitting quantum dots for single-particle tracking and microangiography (*35*, *36*). The clinically approved indocyanine green (ICG) dye also exhibits a weak but detectable SWIR emission (up to ~1500 nm) (*37*). However, much like LOT, DOLI currently relies on extrinsic microparticles and thus lacks efficient and specific labeling strategies for single-cell detection in complex physiological environments.

In this work, we demonstrate efficient labeling of RBCs and neutrophils for single-cell detection and tracking with LOT. The spectrally distinct absorption profiles of different dyes are further exploited to enable multiplexed imaging of these cell populations, an important advance for discerning their dynamic behaviors in vivo. Furthermore, we show that ICG-labeled RBCs can be individually resolved with SWIR cameras, establishing the feasibility of DOLI with clinically approved contrast agents. Last, by coupling SPIONs with the dye labeling approach, we demonstrate simultaneous magnetic control of microcirculation alongside monitoring of single-cell dynamics, further extending this strategy to targeted vascular occlusion in the preclinical ischemic stroke model.

## RESULTS

### Spectrally distinct labeling of RBCs

**Fig. 1.**
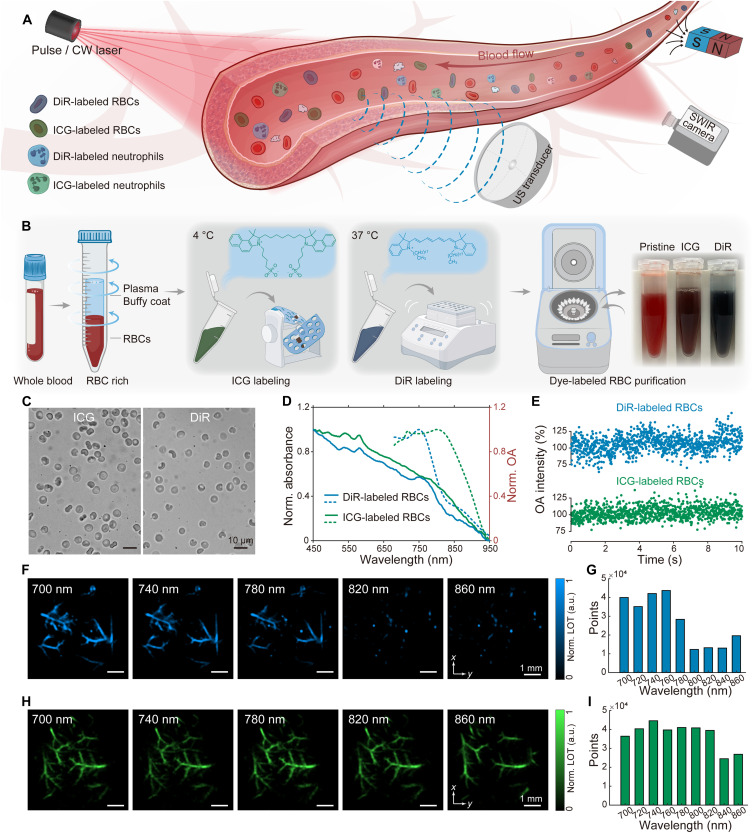
Labeling of blood cells with NIR dyes. (**A**) Conceptual illustration of the envisioned methodology for multiplexed tracking and magnetic actuation of blood cells in vascular networks with OA and FL imaging methods. CW, continuous wave; US, ultrasound. (**B**) Schematic workflow illustrating the labeling of RBCs with ICG or DiR dyes. (**C**) Bright-field microscopy images showing the morphology of labeled RBCs. (**D**) Absorption (solid curves) and OA (dashed curves) spectra of dye-labeled RBCs. (**E**) OA signal stability of dye-labeled RBCs during repeated exposure to 1000 laser pulses. (**F**) MIPs of detected DiR-labeled RBCs in mouse brain vessels, extracted at individual excitation wavelengths (700, 740, 780, 820, and 860 nm) from multispectral image sequences acquired with repeated wavelength cycling between 700 and 860 nm during the imaging session. (**G**) Total number of DiR-labeled RBCs detected at each wavelength. a.u., arbitrary units. (**H**) MIPs of detected ICG-labeled RBCs extracted at the same set of excitation wavelengths from multispectral image sequences acquired under identical conditions. (**I**) Total number of ICG-labeled RBCs detected at each wavelength. a.u., arbitrary units.

The proposed imaging paradigm consists of tagging specific types of blood cells with dyes exhibiting nonoverlapping absorption profiles, creating a multiplexed and multimodal imaging framework leveraging the strengths of both LOT and DOLI (Fig. 1A). For this, blood cells were efficiently labeled with high-density membrane-bound absorbing dyes to enable in vivo single-cell tracking. RBCs isolated from fresh mouse blood were labeled with either ICG or 1,1′-dioctadecyl-3,3,3′,3′-tetramethylindotricarbocyanine iodide (DiR) following optimized protocols, resulting in a visible color change in the cell suspensions (Fig. 1B; see Materials and Methods for details). Bright-field microscopy images revealed that no morphological changes were induced by the labeling procedure (Fig. 1C). Labeled cells exhibited the characteristic absorption peaks of the dyes (Fig. 1D, solid curves), with a sharp peak at ~750 nm for DiR-labeled cells and a broader peak at ~800 nm for ICG-labeled cells. The strong background signal from the cells is partially ascribed to light scattering within the spectrophotometer. The characteristic spectral signatures (absorption peaks) of the labeled cells were further corroborated by OA measurements, where wavelength-scanned OA excitation produced signals unaffected by light scattering that closely matched the absorption profiles (Fig. 1D, dashed curves), confirming successful dye incorporation and strong OA contrast performance. Dye labeling efficiencies reached 89.2 ± 1.9 and 95.1 ± 0.59% for ICG- and DiR-labeled RBCs, corresponding to (1.5 ± 0.29) × 10^8^ and (1.7 ± 0.31) × 10^8^ dye molecules per cell, respectively (fig. S1, A to D). Histogram analysis of single-RBC OA signal intensity revealed a concentrated distribution, indicating relatively homogeneous dye loading across the detected cells (fig. S1, E and G). Repeated exposure to pulsed laser radiation at fluence levels similar to those used in vivo resulted in gradual photobleaching (fig. S2). Yet, the OA signal remained stable for a far longer duration than what is required for image acquisition by the OA imaging system (Fig. 1E). This ensures reliable single-cell localization and tracking with LOT across multiple frames.

Following intravenous injection of 100 μl of cell suspension (~3 × 10^8^ cells/ml), single-cell tracking was achieved with real-time volumetric OA imaging of the murine brain (fig. S3, A and B). Wavelength scanning from 700 to 860 nm in 20-nm increments has further enabled optimized tracking and LOT imaging. Spectral analysis revealed that ICG-labeled RBCs were detectable across a broad wavelength range, while DiR-labeled cells showed no detectable signal at long wavelengths (Fig. 1, F and H). LOT imaging was accordingly possible at wavelengths enabling unambiguous cellular detection. To identify optimal wavelengths for LOT imaging, we quantified detected points exceeding a defined intensity threshold (25% of maximum) across wavelengths (Fig. 1, G and I). The resulting wavelength-dependent cell counts measured in this manner closely matched the absorption spectra of the dyes. On the basis of maximum intensity projections (MIPs) of superimposed dye-labeled cell localizations along the depth direction at each wavelength, vessel maps were generated to extract vascular parameters, including vessel area fraction, total junction number, total vessel length, and end-point count. These parameters exhibited similar wavelength-dependent trends (700 to 860 nm), as described above (fig. S4, A and B). Notably, ICG-labeled cells maintained strong absorption up to 820 nm, suggesting the formation of J-aggregates at the cellular membrane.

### Whole-brain superresolution OA imaging

As natural blood components, RBCs inherently exhibit long circulation times, providing a critical advantage for superresolution imaging that requires tracking numerous individual particles or cells. This becomes particularly valuable when expanding the limited FOV of LOT through array scanning (*31*), as it ensures sustained cell detection across multiple imaging positions.

**Fig. 2.**
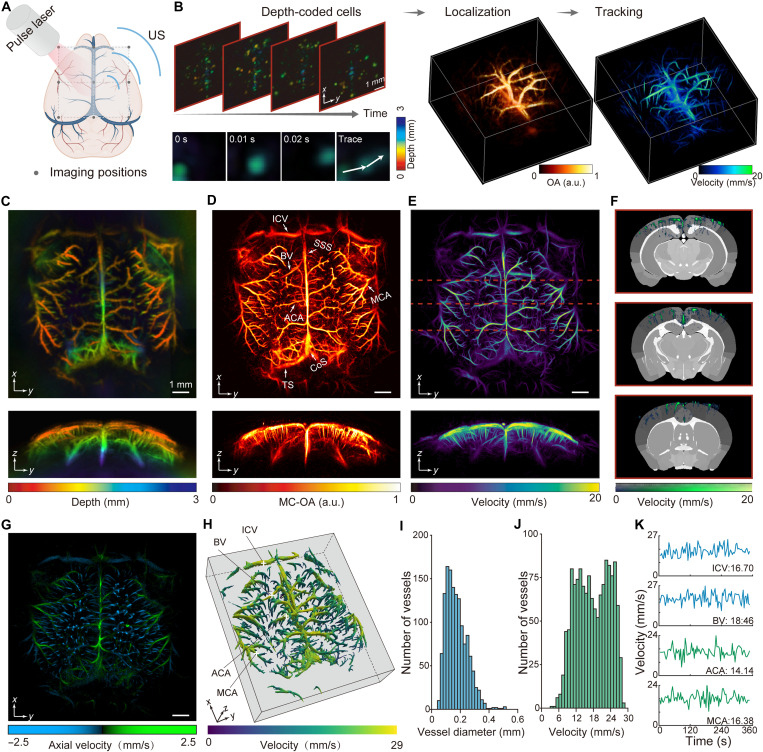
Whole-brain superresolution OA imaging of ICG-labeled RBCs. (**A**) Schematic representation of the scanning protocol covering nine imaging positions for rendering whole-brain images with LOT. (**B**) LOT data processing pipeline illustrating brain vessel reconstruction through localization and tracking of depth-coded ICG-labeled RBCs. (**C**) Whole-brain MC-OA image of the murine brain integrating data from all nine imaging positions color coded for depth. (**D**) Whole-brain LOT image. Anatomical structures labeled: ICV, inferior cerebral veins; BV, bridging veins; SSS, superior sagittal sinus; CoS, confluence of sinuses; TS, transverse sinuses; MCA, middle cerebral artery; ACA, anterior cerebral artery. (**E**) Blood flow VM of the entire cortex. RBC velocities were computed from 3D interframe displacements of tracked cells, yielding instantaneous speed magnitudes in physical units (mm/s). (**F**) Representative coronal brain slices at three anterior-posterior levels marked by a dashed line in (E) display merged atlas-based anatomical segmentation and co-registered velocity information. (**G**) Blood flow direction map based on the axial velocity (*z*-axis component), reflecting the true blood flow velocity along the imaging depth. Positive values denote flow toward the cortical surface, whereas negative values denote flow toward deeper brain layers, shown from a top view of the mouse brain. (**H**) Blood flow velocity overlaid on the segmented vascular network. (**I**) Distribution of the average diameters per vessel segment. (**J**) Distribution of flow velocities per vessel segment. (**K**) Temporal dynamics of flow velocity in four representative vessels as highlighted in (H). Average values are indicated.

To achieve whole-cortex imaging of the murine brain, we implemented a scanning protocol in which the spherical transducer array used to collect OA signals was systematically positioned across a 3 × 3 square grid with 2.5-mm step increments (Fig. 2A). This enabled capturing cell signals from a region spanning the olfactory lobes to the cerebellum (fig. S5). The laser wavelength was tuned to 800 nm, corresponding to the absorption peak of the dye and also to the wavelengths for which more cells were detected in the multispectral imaging experiment described above. LOT operates on the principle of detecting individual strong absorbers within a sequence of OA frames. Given the intrinsic 3D imaging capability of the OA tomographic system used, depth-resolved detection of isolated absorbers was possible for a depth range of ~3.2 mm following singular value decomposition (SVD)–based filtering to suppress stationary tissue background signals (Fig. 2B and movie S1). 3D LOT images could then be generated by accumulating the positions of localized points (Fig. 2B), revealing vascular networks that remained undetectable with conventional OA tomography despite the intrinsic contrast of RBCs. In addition, by tracking the localized positions in consecutive frames, it was possible to quantify interframe displacements and thus build a velocity map (VM) for the resolved microvasculature (Fig. 2B).

Additional processing and compounding of images from all scanning positions enabled generation of multiple image types, providing a comprehensive assessment of the murine cortical vasculature. So-called motion-contrast OA (MC-OA) images can be generated by compounding images of flowing RBCs in the sequence of frames obtained after SVD filtering (Fig. 2C). MC-OA images have the same resolution as the original OA images but provide a substantially enhanced view of vascular networks and further mitigate so-called limited-view artifacts (*31*). MIPs of the MC-OA image of the scanned region color coded for depth provide a comprehensive view of the volume being covered (Fig. 2C). The LOT image of the entire cortex can also be computed and enhanced with histogram equalization of the MIP views, revealing otherwise invisible microvascular details (Fig. 2D). Likewise, the VM of the scanned region provides a holistic view of cortical blood flow (Fig. 2E), while co-registration of the entire VM with the standard brain atlas enables identification of flow velocity in pial and penetrating vessels in specific regions, as illustrated with three representative overlapping slices from anterior, middle, and posterior regions (Fig. 2F) (*38*). Complementarily, two rotating 3D renderings of MC-OA and VM are provided in movie S2 to illustrate the volumetric distribution of cerebral vasculature and flow velocity. In addition, incorporating flow direction information into the LOT images enables discrimination between vessels carrying blood from the cortical surface into the deeper layers and those exhibiting the opposite flow orientation (Fig. 2G). By applying image thresholding and morphological cleaning, a segmentation map was generated, from which a vascular graph with edge-wise diameter and flow velocity information was obtained (Fig. 2H) (*39*, *40*). Quantitative analysis of the segmented network yielded vessel diameter and flow velocity distributions, enabling comprehensive morphological and functional characterization of the LOT data (Fig. 2, I and J). Temporal profiles of flow velocity variations in representative arteries and veins further demonstrate the ability of the proposed approach to extract quantitative hemodynamic information from the brain with a temporal resolution of 4 s (Fig. 2K).

### Multiplexed OA imaging of blood cell subtypes

**Fig. 3.**
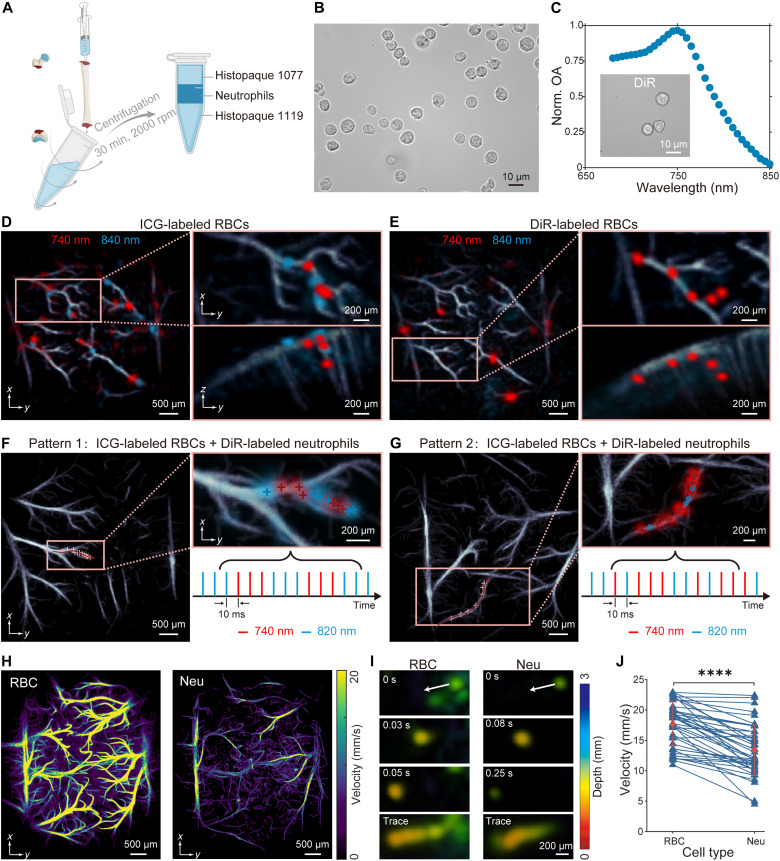
Multiplexed OA imaging of spectrally distinctive labeled RBCs and neutrophils. (**A**) Workflow for neutrophil extraction from the murine bone marrow. (**B**) Bright-field microscopy image of extracted neutrophils. (**C**) OA spectrum of DiR-labeled neutrophils with an inserted bright-field image showing cellular morphology. (**D**) LOT images rendered with localized points of superimposed ICG-labeled RBCs acquired at two consecutive wavelengths (740 and 840 nm). Zoom-in *xy* and *yz* views are shown across sequential frames. (**E**) LOT images rendered with localized points of superimposed DiR-labeled RBCs acquired at two consecutive wavelengths (740 and 840 nm). Zoom-in *xy* and *yz* views are shown across sequential frames. (**F**) LOT images rendered with detected tracks of mixed ICG-labeled RBCs and DiR-labeled neutrophils, illustrating tracking of ICG-labeled RBCs within a pulse sequence of three consecutive 740-nm excitations followed by three consecutive 820-nm excitations. (**G**) LOT images rendered with detected tracks of mixed ICG-labeled RBCs and DiR-labeled neutrophils, illustrating tracking of DiR-labeled neutrophils within a pulse sequence of 1- by 740-nm, 1- by 820-nm, 3- by 740-nm, and 2- by 820-nm excitations. (**H**) Representative VMs of dye-labeled RBCs and neutrophils in the same mouse. (**I**) Representative frames at selected time points showing the motion of RBCs (left) and neutrophils (right), with trajectories overlaid in the final frame. (**J**) Paired comparison of average velocities between dye-labeled RBCs and neutrophils within the same vessel segments. Data are from 3 mice and 42 ROIs (****P < 0.0001, paired *t* test).

OA excels for its unique capability to unambiguously discriminate multiple biomolecules, cell types, or contrast agents on the basis of distinct absorption spectra (*41*). This can open unprecedented capabilities for multiplexed blood cell imaging, i.e., simultaneous visualization of different circulating cell populations in a single acquisition without compromising the spatiotemporal resolution. In addition to RBCs, neutrophils isolated from the bone marrow were also labeled with DiR and ICG (Fig. 3, A and B; see Materials and Methods for details). Similar to RBC labeling, dye-labeled neutrophils retained their intact morphology and exhibited OA spectral signatures consistent with the respective dyes (Fig. 3C). ICG- and DiR-labeled neutrophils exhibited labeling efficiencies of 64.8 ± 5.2 and 71.5 ± 7.0%, corresponding to (3.7 ± 0.21) × 10^9^ and (2.4 ± 0.79) × 10^10^ dye molecules per cell, respectively (fig. S1, A to D). Single-cell OA intensity histograms showed relatively narrow distributions (fig. S1, F and H). Across both DiR and ICG labeling conditions, neutrophils consistently exhibited lower mean OA intensities than their RBC counterparts. This difference may be attributed to the intrinsic hemoglobin absorption in RBCs, which augments the overall OA response. Single-cell neutrophil dynamics were captured following intravenous injection of the labeled suspension (100 μl, ~5 × 10^7^ cells/ml), with concurrent multiwavelength (700 to 860 nm, 20-nm step) OA imaging of the murine brain. Much like for labeled RBCs, LOT imaging of labeled neutrophils was feasible at wavelengths corresponding to strong absorption of ICG and DiR, with wavelength-dependent cell counts and vessel quantification closely matching the absorption spectra of the dyes (figs. S3, C and D; S4, C and D; and S6).

Multiplexed imaging of cells labeled with different dyes was enabled by exploiting the differential absorption profiles of ICG and DiR. Two wavelengths were selected so that ICG-labeled cells exhibit absorption at both, whereas DiR-labeled cells absorbed only at one. This spectral distinction facilitated the selective visualization of each cell population in the OA images. Note that the laser system used for OA imaging allows per-pulse tuning at 100 Hz within the NIR range, enabling acquisition of OA images at 50 frames per second for alternating wavelengths, a rate sufficient for tracking cells in the bloodstream. To demonstrate spectral discrimination in sequential OA images, we separately injected DiR- and ICG-labeled RBCs intravenously and imaged the murine brain while alternating between 740- and 840-nm excitation wavelengths. Superimposed SVD-filtered OA images reveal that ICG-labeled cells are visible in consecutive frames at both 740 and 840 nm (Fig. 3D) so that single-cell tracking is facilitated by the fact that small interframe distances can be detected. On the contrary, DiR-labeled RBCs were exclusively detectable at 740 nm (Fig. 3E). Even in vessels with high blood velocity where cells appear only in alternating frames, single-cell tracking remains feasible because of their distinct spectral signature.

Multiplexed in vivo cell imaging was validated by intravenous injection of a 100-μl mixture containing ICG-labeled RBCs and DiR-labeled neutrophils, where OA distinguished the populations via sequential wavelength excitation and single-cell tracking. Specific wavelength sequences were defined for this purpose. First, a sequence consisted of alternating three pulses at 740 nm and three pulses at 820 nm (Fig. 3F). Tracking was performed with zero-gap enforcement, i.e., considering only trajectories where cells appeared in consecutive frames without interruption. ICG-labeled RBCs were identified as valid tracks comprising three sequential detection points at 820 nm (Fig. 3F). Gap-free tracking enabled reliable discrimination between flowing RBCs and stationary noise sources randomly appearing as dots in the acquired sequence of images. DiR-labeled neutrophils were differentiated using a distinct wavelength sequence that defined a characteristic pattern (Fig. 3G). In this case, the tracking algorithm permitted a two-frame gap, accepting trajectories even with intermittent detection. The number of wavelengths defining the pattern was optimized so that typical cell transit times in the FOV exceed the pattern length while remaining sufficiently long for reliable identification. DiR-labeled cells were identified by their characteristic excitation pattern, contrasting with the consecutive-frame appearances of ICG-labeled RBCs. VMs reconstructed from the same brain region in the same mouse revealed distinct flow dynamics, with neutrophils exhibiting markedly slower motion compared to RBCs (Fig. 3, H and I). Quantification across multiple vessel segments showed significantly lower mean velocities for neutrophils than for RBCs (Fig. 3J).

### Capillary-level microangiography in the second NIR window

ICG is primarily used for its strong FL and OA contrast in the first NIR region (NIR-I; 700 to 900 nm). However, it also exhibits a weak but consistent emission tail extending to ~1500 nm covering the NIR-II window (*37*, *42*). This secondary emission, although orders of magnitude lower in intensity than its NIR-I peak, has been proved to be detectable with sensitive SWIR cameras and spectral unmixing techniques. Critically, this property can enable dual OA-FL imaging of ICG-labeled cells with a contrast agent that is US Food and Drug Administration approved.

**Fig. 4.**
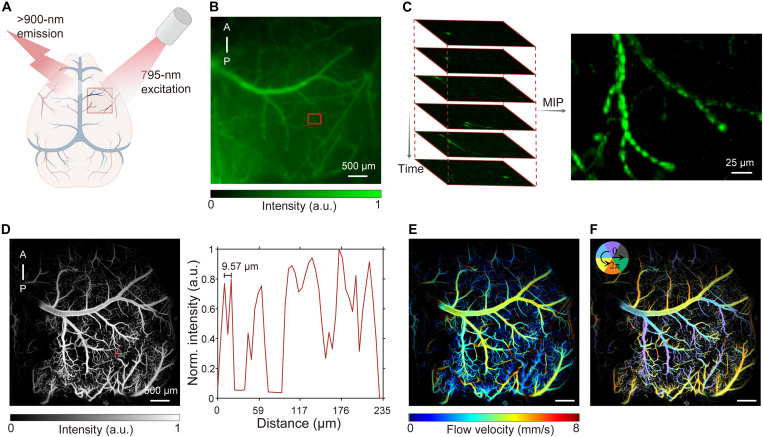
FL localization imaging of ICG-labeled RBCs in the NIR-II window. (**A**) Schematic representation showing the cortical vessel area being imaged. (**B**) NIR-II wide-field FL imaging of cortical vessels acquired 1 min after intravenous injection of ICG-stained RBCs. (**C**) FL detection of individual ICG-labeled cells in a time sequence within the red dashed rectangle in (B), acquired 40 min postinjection, and corresponding MIP image with background removal. (**D**) Structural map of the murine brain reconstructed with FL localization imaging. The line signal profile along the red line is shown on the right. (**E**) Color-coded flow VM. (**F**) Color-coded flow direction map. (D to F) Scale bar, 500 μm.

To validate NIR-II DOLI with ICG-labeled RBCs, we implemented cranial window preparation in C57BL/6J wild-type mice using an established surgical protocol (Fig. 4A; see Materials and Methods for details). Labeled blood cells, circulating within the vasculature, act as point emitters appearing as dots in raw SWIR images. This behavior was first confirmed by flushing a suspension of labeled cells through a tubing, where individual flowing cells were directly visualized (fig. S7 and movies S3 to S5). Single emitters began to be detected in vivo within the cranial window region starting 40 min after intravenous injection (100 μl of bolus) and appeared more prominent after SVD filtering of the image sequence (Fig. 4, B and C). Representative FL image sequences acquired after 1 min and at 40 min postinjection are shown in movie S6. These findings confirm the prolonged circulation time of dye-labeled cells and benefit long-term dynamic imaging.

Much like for LOT, the accumulated positions of localized points corresponding to isolated cells can be superimposed to reconstruct the DOLI image (Fig. 4D), which enables achieving resolution beyond that of the original wide-field SWIR images (*35*). Note that the SWIR camera provides markedly higher resolution than OA tomography, although this deteriorates with depth because of light scattering. A high wide-field resolution facilitates resolving finer details in microvascular networks down to the capillary level, particularly in superficial vessels resolved with ballistic photons (fig. S8). Tracking of ICG-labeled RBCs further allows for velocity mapping down to the finest microvascular networks as well as determination of flow direction (Fig. 4, E and F), thereby enabling precise characterization of microvascular dynamics. Although DiR does not provide longer-wavelength emissions in the NIR-II window, DiR-labeled RBCs were nevertheless successfully applied for localization-based FL imaging of the cerebrovascular network through intact skull upon 750-nm excitation and collection of the emitted light above 775 nm within its emission spectrum (fig. S9). The observed difference in performance is likely attributable to the use of the NIR-II tail emission of ICG for imaging and the higher readout noise of the SWIR detector.

### OA monitoring of magnetically actuated, dye-labeled blood cells

**Fig. 5.**
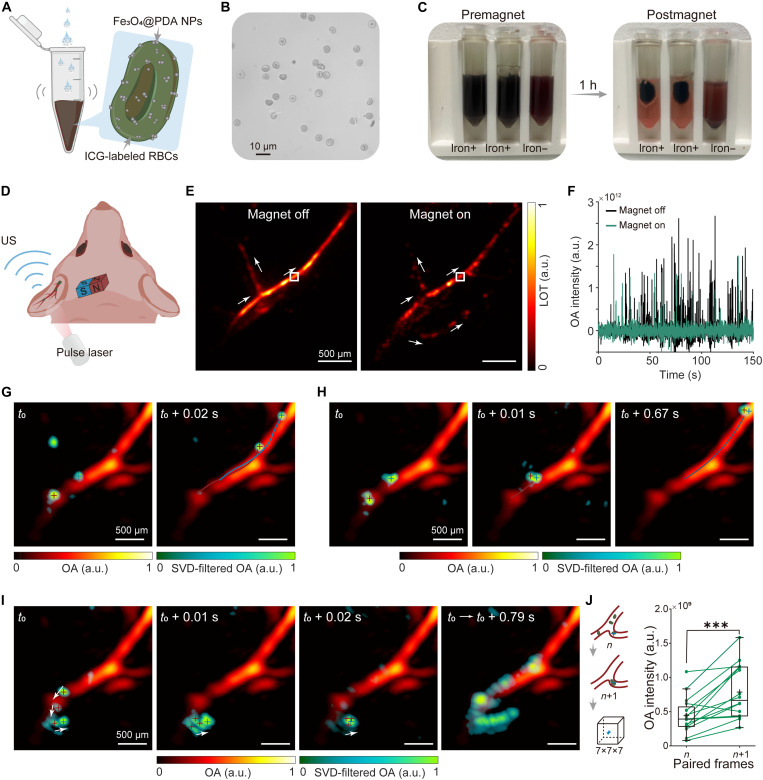
Magnetic actuation of ICG-labeled RBCs in vitro and in vivo. (**A**) Schematic of Fe_3_O_4_@PDA-modified RBC preparation. (**B**) Bright-field microscopy images showing the morphology of ICG-labeled RBCs after Fe_3_O_4_@PDA NPs loading. (**C**) Photographs of suspensions of ICG-labeled RBCs with or without Fe_3_O_4_@PDA NPs exposed to an external magnet. h, hour. (**D**) Schematic of in vivo OA monitoring of dye-labeled RBCs under magnetic actuation. (**E**) LOT images of the same vascular region with and without a magnetic field. (**F**) Temporal profiles of the SVD-filtered OA signals in the main vessel marked by the white square before and after magnet application. (**G**) SVD-filtered images overlaid with OA vessel maps showing the normal flow of magnetic RBCs without a magnet. (**H**) SVD-filtered images showing cell aggregation and slow movement under a magnet. (**I**) SVD-filtered images showing RBCs redirected from the main vessel to a branch under magnetic actuation. (**J**) Frame-pair analysis of OA signals at the branch point and corresponding quantitative comparison (***P = 0.0003, paired *t* test). White arrows indicate the blood flow direction, while green dots mark the individual cells. Colored plus symbols indicate the localization of the same signal point in different frames, and the corresponding-colored lines depict its trajectory over time.

To impart magnetic functionality to dye-labeled blood cells, SPIONs coated with polydopamine [polydopamine-coated iron oxide nanoparticles (Fe_3_O_4_@PDA NPs)] were introduced via a straightforward autopolymerization reaction under alkaline conditions (*32*, *43*). Owing to the strong adhesion properties of the PDA shell, Fe_3_O_4_@PDA NPs readily attached to ICG-labeled RBCs through simple mixing (*44*) while preserving native cellular morphology and good deformability (Fig. 5, A and B, and fig. S10). Notably, modified cells aggregated and remained in suspension near the region exposed to the magnetic field, whereas cells lacking Fe_3_O_4_@PDA NPs underwent gravitational sedimentation to the bottom of the tube (Fig. 5C).

The feasibility of in vivo magnetic actuation was evaluated by intravenous injection of 100 μl of magnetic ICG-labeled RBCs, followed by OA imaging of the mouse ear vasculature with the magnet moved manually (Fig. 5D). In the absence of a magnet, LOT revealed clear vascular structures, including one main vessel and a branch vessel (Fig. 5E). A previously unseen branching vessel appeared under magnetic actuation, while some original vascular structures lost clarity (Fig. 5E). Given that LOT reconstruction relies on localizing and tracking individual absorbers, the integrity of vascular structures depends on the number of dye-labeled cells circulating in the vessels. To further characterize labeled cell flow, SVD filtering was applied to raw OA images to eliminate low-frequency background fluctuations and high-frequency noise from blood. Under magnetic influence, the temporal profile of filtered OA signals in a region of the main vessel exhibited a marked reduction in the number of localized signal events (Fig. 5F). In the FOV, cells typically flowed along the vessel within ~0.03 s when passively following the blood flow (Fig. 5G). In contrast, upon application of the magnet, the cells aggregated, and it required ~0.67 s for them to traverse the same vessel length (Fig. 5H). Side-by-side image sequences illustrate fast cell motion without magnetic actuation (left) and slower motion with magnetic actuation (right) (movie S7). This behavior likely arises from magnetic forces acting on modified cells, either directly opposing blood flow or indirectly increasing hydrodynamic drag through transient clustering. Notably, magnetic actuation caused some cells to remain at a vessel branch point and simultaneously forced cells from the main vessel to change direction and aggregate at the same site (Fig. 5I). Quantitative analysis of numerous frame pairs was conducted, with the first frame showing cells in both the main vessel and branch point and the second frame showing cells confined to the branch point. OA signal intensities measured at the fixed branch location consistently demonstrated a significant enhancement in the second frame, indicating that magnetic actuation redirected a fraction of cells from the main vessel toward the branch point (Fig. 5J). The formed cell clusters did not remain permanently at the branch point. Rather, they were subsequently guided into the side branch and continued flowing along it. This dynamic redistribution accounted for the appearance of a previously unseen vessel in the LOT image. In addition, in vivo experiments with magnetic DiR-labeled RBCs revealed that most cells preferentially flowed within the main vessel, leading to incomplete reconstruction of small vessels. This further confirms that external magnetic fields can alter the distribution of labeled cells within the microvasculature (fig. S11). Taken together, the findings establish OA imaging as a feasible method for real-time tracking of dye-labeled blood cells under magnetic actuation. Magnetically guided cell delivery emerges as a promising strategy, allowing revisualization of hidden vessels and precise targeting within the vascular network.

### Magnetically induced vessel occlusion

**Fig. 6.**
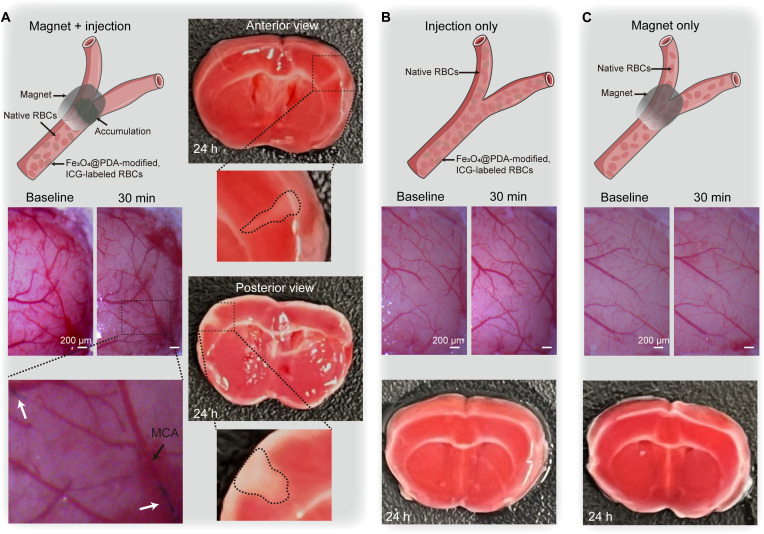
Magnetically induced vascular occlusion model. (**A** to **C**) Schematic representations of the experimental procedure together with representative bright-field images and coronal slices stained with TTC from mice subjected to different experimental conditions: intravenous injection of Fe_3_O_4_@PDA-modified, ICG-labeled RBCs following magnet placement (magnet + injection) (A); intravenous injection of Fe_3_O_4_@PDA-modified, ICG-labeled RBCs only (B); and magnet placement only (C). White arrows indicate vessels with evident occlusion in the bright-field images. Black dashed outlines delineate the lesion area in the TTC images and the corresponding enlarged views.

In addition to cell tracking, we investigated whether the magnetic ICG-labeled RBCs can be controlled to induce a precise vascular occlusion. To test the feasibility of in vivo magnetic manipulation of Fe_3_O_4_@PDA-modified, ICG-labeled RBCs, we applied magnetic forces to the middle cerebral artery (MCA) region, one of the main arteries supplying the somatosensory cortex. Under an external magnetic field, mice injected with modified RBCs exhibited pronounced localized retention and aggregation of cells within cortical microvessels, resulting in evident vascular occlusion, as well as the formation of clearly defined infarcts in the ipsilateral cortex, while no detectable damage was observed in the contralateral hemisphere (Fig. 6A). The occlusion is reversible, i.e., the vessel was recanalized when the magnet was retracted. In contrast, neither intravenous injection of engineered cells alone nor placement of the external magnet alone produced detectable vascular obstruction or apparent tissue damage (Fig. 6, B and C). These findings demonstrate that magnetic targeting of modified cells can effectively induce precise and reversible vascular occlusion, thereby establishing a controllable and spatially confined model of focal stroke.

## DISCUSSION

In vivo cell tracking plays a pivotal role across multiple biological and clinical research areas. The ability to image labeled cells has profoundly advanced our understanding of key biological processes and therapeutic interventions, especially in stem cell therapies, oncology, and immunology (*45*, *46*). Various molecules and nanoparticles have been used to label cells for whole-body imaging modalities such as x-ray computed tomography (CT) and MRI. For instance, SPIONs enable MRI-based tracking of stem cells (*47*, *48*), while gold nanoparticles support CT imaging of immune cells (*49*, *50*). Nuclear imaging techniques such as PET and SPECT facilitate cell tracking via long-lived radioisotope labeling (*51*). This enables prolonged imaging windows, although at the cost of reduced temporal resolution. Optical imaging techniques, including bioluminescence and FL, have also demonstrated robust efficacy for tracking cells across diverse anatomical sites in preclinical models (*52*), but their spatial resolution is constrained by light scattering. Emerging imaging modalities are further unlocking new possibilities for cell tracking. Magnetic particle imaging offers superior sensitivity compared to MRI for detecting SPION-labeled cells, though current detection thresholds remain limited to several thousand cells (*53*). On the other hand, multispectral OA tomography has surpassed x-ray CT in terms of sensitivity when tracking cells labeled with gold nanorods (*54*). OA contrast is also particularly versatile, enabling robust cell detection through exogenous contrast agents or genetic reporters (*55*–*58*). Ultrasound has also advanced for cellular imaging thanks to the emergence of genetically encoded gas vesicles serving as effective pulse-echo contrast agents (*21*, *59*).

Tracking individual RBCs could shed new light into microvascular pathologies (e.g., sickle cell disease), while leukocyte monitoring may reveal immune recruitment during inflammation, and detection of rare circulating melanoma tumor cells could clarify metastatic mechanisms (*60*–*62*). However, despite substantial progress in deep-tissue cell tracking, existing approaches remain limited in sensitivity and multiplexing capacity and thus fall short of robust single-cell detection. The present work establishes a biocompatible framework for NIR OA and FL labeling of RBCs and neutrophils and deep-tissue single-cell tracking with localization-based imaging. Leveraging spectrally distinct absorption profiles further enables multiplexed imaging of different cell populations, resolving their in vivo dynamics with a high spatiotemporal resolution while also uncovering differences in the velocities of different cell types within opaque tissues. In addition, coupling SPIONs with optical labels introduces magnetic control alongside real-time monitoring of microcirculatory dynamics. Prolonged magnetic field exposure enhances magnetic forces acting on circulating cells, promoting their localized retention and aggregation within microvessels and therefore enabling controllable induction of focal ischemic stroke. Together, this multimode platform, combining multiplexed high-resolution imaging with targeted cell manipulation, provides a versatile strategy for investigating microvascular physiology and pathology at the single-cell level using clinically translatable contrast agents.

By leveraging localization and tracking of individual cells labeled with ICG, a dye approved for clinical use since 1956, LOT and DOLI provide a powerful strategy for microvascular imaging, achieving spatial resolutions beyond conventional acoustic diffraction and optical diffusion limits and presenting a promising pathway for clinical translation. Unlike conventional vascular imaging modalities, localization-based imaging relies on the precise detection of isolated point absorbers rather than structural continuity, thus rendering spatial precision largely independent of vessel orientation and inherently less susceptible to limited-view artifacts (*28*). On the basis of its in vivo performance, LOT achieves lateral and axial resolutions down to 24 and 32 μm, respectively, although the resolution gradually decreases with depth (table S1). DOLI further enhances the spatial resolving capacity, enabling visualization at a capillary level. The effective temporal resolution of LOT is currently ~4 s for reconstructed velocity mapping, constrained by the laser pulse repetition frequency (100 Hz), which is lower than that of state-of-the-art ultrasound localization microscopy (ULM) implementations (*63*). ULM can also reach larger depths yet lacks the multiplexing capacity and sensitivity achieved with optical contrast. Future improvements in laser repetition rates and computational strategies for sparse acquisition, including deep neural network–based reconstruction (*64*), may substantially speed up image acquisitions. This is also particularly important for expanding the biological applications of multiplexed imaging of RBCs and neutrophils. Although multiphoton or light sheet microscopy methods allow for simultaneous multicolor imaging of cells and vascular structures in vivo (*65*, *66*), these methods require tighter trade-offs between imaging depth, speed, and FOV. In contrast, LOT and DOLI enable noninvasive imaging of living tissues over a larger FOV, allowing interactions among diverse cell populations to be observed under physiologically intact conditions and thereby more faithfully reflecting native biological processes. Multiplexed imaging relies on tracking of moving cells, which is challenged by signal intensity changes associated with differences in acoustic sensitivity and light fluence. The tracking algorithm may also fail in vessels exhibiting fast flows, which may result in large interframe distances, particularly if a gap between frames is allowed, and false negative/positive track assignments.

In addition, integration with clinically approved SPIONs further extends the functionality of the platform. By modulating the magnetic field, Fe_3_O_4_@PDA-modified RBCs exhibit controllable magnetic responses. The capability for magnetic actuation of cells, specifically to induce controlled aggregation, has important implications as it enables the occlusion of blood flow in microvessels. Under short and moderate magnetic conditions, cells can be redistributed or guided to accumulate within specific vascular regions, providing a potential foundation for targeted delivery strategies. In contrast, prolonged or intensified magnetic exposure promotes sustained cellular aggregation within microvessels, resulting in localized vascular occlusion, as demonstrated here for inducible focal ischemic stroke. Furthermore, the combination of magnetic manipulation of engineered cells with simultaneous LOT-based visualization of cerebrovascular structural changes provides a strategy for noninvasive stroke model induction while enabling real-time confirmation of successful model establishment. Despite these promising capabilities, the long-term biological fate of SPION-functionalized cells and their interactions with the microvascular environment remain to be investigated. An improved understanding of these aspects may broaden the potential for engineering cells labeled with clinically translatable agents and integrating them with advanced imaging modalities for both mechanistic studies and therapeutic applications.

## MATERIALS AND METHODS

### Materials

d-(+)-Glucose, adenine, citric acid monohydrate (C_6_H_8_O_7_·H_2_O), sodium phosphate monobasic monohydrate (NaH_2_PO_4_·H_2_O), sodium citrate tribasic dihydrate (Na_3_C_6_H_5_O_7_·2H_2_O), ethylenediaminetetraacetic acid (EDTA), dopamine hydrochloride (DA), Fe_3_O_4_ NPs (cat. no. 900043; size: 15 nm), 2,3,5-triphenyltetrazolium chloride (TTC; cat. no. T8877), ATX tris buffer, Histopaque 1077, Histopaque 1119, Dulbecco’s phosphate-buffered saline (DPBS), and Diluent C were purchased from Sigma-Aldrich (St. Louis, MO, US). ICG was obtained from Tokyo Chemical Industry Co., Ltd. (Tokyo, Japan). XenoLight DiR fluorescent dye was purchased from PerkinElmer (Waltham, MA, US). Sodium chloride (NaCl), RPMI 1640 medium, fetal bovine serum (FBS), and phosphate-buffered saline (PBS) were purchased from Thermo Fisher Scientific (Waltham, MA, US). All experiments were performed using double-deionized water (resistivity, 18.2 MΩ·cm) generated by a Milli-Q EQ7000 water purification system (Millipore, Billerica, MA, US).

### Animal and ethics

The animal experiments were performed in compliance with the Swiss Federal Act on Animal Protection and were approved by the Cantonal Veterinary Office Zurich (ZH060/2022 and ZH030/2023). Foxn1nu nude mice (4 to 6 weeks old, female, Envigo BMS B.V., The Netherlands) and C57BL/6J mice (4 to 6 weeks old, female, Charles River Laboratories, Germany) were used for imaging experiments. The mice were housed in individually ventilated cages under standard laboratory conditions (12-hour light/dark cycle, temperature maintained at 22°C, and relative humidity of ~50%), with ad libitum access to food and water. For imaging, mice were anesthetized with isoflurane [5% (v/v) for induction and 1.5% (v/v) for maintenance, Abbott, Cham, Switzerland] delivered in an oxygen/air mixture (200/800 ml/min for induction and 100/400 ml/min for maintenance). For cranial window experiments, mice were positioned in a stereotactic frame with rounded ear bars and maintained on a warming mat (36°C). Prophylactic analgesia was administered via subcutaneous buprenorphine (2 μl/g body weight) 30 min before surgery. The scalp was disinfected with Betadine and 70% ethanol, followed by a small incision to expose the skull. A rectangular region of the skull overlying the MCA was then marked and carefully removed using a microdrill. Subsequently, a sterile glass coverslip was fixed to create a stable optical window for imaging.

### RBC extraction and labeling

Fresh whole blood was collected via cardiac puncture after euthanizing the mouse into Microtainer tubes (cat. no. 363705, Becton Dickinson, US) and centrifuged at 800 rpm (60*g*) for 7 min (Centrifuge 5415 D, Eppendorf, Germany) to remove plasma and buffy coat.

#### ICG-labeled RBCs

The extracted blood was washed three times with nutrient-additive solution AS-3, which was used for staining RBCs to preserve their viability and structural integrity during prolonged incubation. The RBCs were then diluted to 20% hematocrit in AS-3. A 0.5-ml aliquot of this suspension was incubated with 50 μl of ICG stock solution (2 mg/ml) under gentle end-over-end rotation (30 rpm) using a tube rotator for 1.5 hours at 4°C. Unbound dye was removed by four successive washes with AS-3 via centrifugation at 500 rpm (27*g*) for 15 min (Centrifuge 5430 R, Eppendorf, Germany). The final pellet was resuspended in 100 μl of AS-3, yielding a working concentration of ~3 × 10^8^ cells/ml, suitable for sparse-cell imaging in vivo (*67*).

#### DiR-labeled RBCs

The extracted blood was washed three times with 1× DPBS without Ca^2+^/Mg^2+^ at 800 rpm (60*g*) for 7 min at 37°C to avoid potential interference with the DiR staining process. The isolated RBCs were diluted to 25% hematocrit in DPBS. For labeling, 0.3 ml of this suspension was resuspended in 0.9 ml of DPBS. A DiR labeling solution was prepared by diluting 9 μl of DiR stock solution (20 mg/ml in 100% ethanol) into 1.2 ml of Diluent C. The staining mixture was incubated at 37°C for 10 min using a Matrix Orbital Thermoshaker (model F2.0, IKA, Staufen, Germany) set to 300 rpm. After incubation, excess dye was removed by four washes with DPBS (800 rpm = 60*g*, 7 min), and the final pellet was resuspended in 100 μl of DPBS to a final concentration of ~3 × 10^8^ cells/ml for subsequent in vivo imaging.

### Neutrophil isolation and labeling

Neutrophils were isolated according to a previously reported method (*68*). Briefly, femurs and tibias were harvested from euthanized mice, cleaned of surrounding muscle tissue, and disinfected with 70% ethanol. Bone marrow was flushed out using a 26-gauge needle connected to a 1-ml syringe filled with RPMI 1640 medium supplemented with 10% FBS and 2 mM EDTA. The cell suspension was filtered through a 100-μm cell strainer into a 50-ml screw-cap Falcon tube and centrifuged at 1400 rpm for 7 min at 4°C. RBCs were lysed by resuspending the pellet in 20 ml of 0.2% NaCl for ~20 s, followed by the immediate addition of 20 ml of 1.6% NaCl. After centrifugation, the bone marrow cells were resuspended in 1 ml of ice-cold PBS and carefully layered over a two-step Histopaque gradient consisting of 3 ml of Histopaque 1077 (density: 1.077 g/ml) layered on top of 3 ml of Histopaque 1119 (density: 1.119 g/ml) in a 15-ml Falcon tube. The gradient was centrifuged at 2000 rpm for 30 min at 25°C without brake. Neutrophils were collected from the interface between the two layers, washed twice with RPMI-1640 containing 10% FBS and 1% penicillin/streptomycin, and centrifuged at 1400 rpm for 7 min at 4°C.

#### ICG-labeled neutrophils

Neutrophils (0.5 ml) were mixed with 0.5 ml of AS-3 supplemented with 75 μl of ICG stock solution (2 mg/ml) and incubated for 1 hour at 4°C under gentle end-over-end agitation (30 rpm). The labeled cells were washed, pelleted by centrifugation, and resuspended to a concentration of ~5 × 10^7^ cells/ml.

#### DiR-labeled neutrophils

Neutrophils (0.6 ml) were combined with 0.6 ml of DPBS and 4 μl of DiR stock solution (20 mg/ml) and incubated at 37°C for 15 min under orbital shaking at 300 rpm. Excess dye was removed by four washes with DPBS, and the labeled pellet was resuspended to a concentration of ~5 × 10^7^ cells/ml for further use.

### Generation of SPION-functionalized dye-labeled blood cells

The magnetic functionality of dye-labeled blood cells was introduced by incorporating Fe_3_O_4_@PDA NPs. Briefly, 250 μl of Fe_3_O_4_ NPs (5 mg/ml in water) and 100 μl of DA (1 mg/ml in tris buffer) were rapidly added to a solvent mixture of 4.75 ml of deionized water and 4 ml of tris buffer. The reaction mixture was gently stirred at room temperature for 3 hours. Because of the alkaline conditions provided by tris buffer, DA underwent auto-oxidation and polymerization, forming a polydopamine shell around the Fe_3_O_4_ NPs (*32*, *43*). The resulting Fe_3_O_4_@PDA NPs were purified by centrifugation using Amicon Ultracentrifugal filters (10-kDa molecular weight cutoff) at 4000 rpm for 30 min (Centrifuge 5430 R, Eppendorf, Germany) and collected in 500 μl of water. Subsequently, 75 μl of Fe_3_O_4_@PDA NPs was mixed with the previously prepared dye-labeled blood cells and incubated under gentle agitation for 1.5 hours. The magnetic cells were then washed, pelleted by centrifugation, and resuspended to a final concentration of ~3 × 10^8^ cells/ml.

### Characterization

The morphology of labeled cells was observed and recorded using a bright-field microscope (Primo Star, ZEISS, Germany) equipped with a Basler ace 2 Basic camera (model a2A2590-60umBAS, Basler AG, Germany). The absorption spectra of labeled cells were measured with an AVASPEC spectrometer (Avantes, The Netherlands). Dye labeling efficiency and the number of dye molecules per cell were estimated by establishing a standard calibration curve of optical absorbance versus known dye concentrations, quantifying the free dye remaining in the collected supernatant after purification, and determining the number of recovered cells using a hemocytometer.

The OA spectra of the labeled cells were acquired using a custom-built OA spectroscopy system. A 256-element spherical array transducer (Imasonic SaS) with a central frequency of 4 MHz was positioned in an upward-facing configuration, with its internal cavity filled with 1% agar gel to provide optimal acoustic coupling. A 5-μl labeled cell suspension was dropped onto a glass coverslip placed atop the gel surface. Multispectral illumination was provided from above using a tunable nanosecond-pulsed optical parametric oscillator (OPO) laser (model: SpitLight EVO II OPO-532, Innolas GmbH, Krailling, Germany) coupled to a fiber bundle, delivering excitation light in 5-nm increments across the 680- to 950-nm wavelength range. OA signals were recorded at each wavelength, and the corresponding spectral profiles were extracted using MATLAB R2022a by quantifying the wavelength-dependent signal. To correct for wavelength-dependent fluence variations of the OPO output, all signals were normalized to those obtained from a reference ink solution measured under identical conditions. To assess the uniformity of dye labeling, a polyethylene tubing (inner diameter, 0.28 mm; outer diameter, 0.61 mm) was embedded in 1% agar within the region excited with the pulsed laser, and OA signals from individual sparse dye-labeled cells flowing through the tubing, driven by slow syringe infusion, were captured and quantified. To evaluate the effect of pulsed laser exposure on the OA signal, ICG- and DiR-labeled RBCs were subjected to 1000 laser pulses at an energy level corresponding to in vivo imaging conditions. For OA signal stability assessment, ICG-labeled RBCs were irradiated for 30, 180, and 360 s at a pulse energy of 18 mJ/cm^2^. For FL signal stability, ICG-labeled RBCs were exposed to pulsed laser irradiation for 60 s at two energy levels, 26 and 18 mJ/cm^2^, respectively.

### OA imaging system

OA tomographic imaging of the cerebral vasculature was conducted on anesthetized mice using a custom-built OA tomography system. Nude mice were used to allow fully noninvasive imaging without fur removal. Animals were positioned prone on a heating pad maintained at 37°C, with the head fixed in a stereotactic frame to minimize motion. The eyes were protected with dexpanthenol cream. A 100-μl suspension of the labeled cells (RBCs: ∼3 × 10^8^ cells/ml; neutrophils: ∼5 × 10^7^ cells/ml) was administered intravenously via the tail vein. The imaging setup featured a 512-element piezoelectric spherical array transducer (Imasonic SaS, Voray, France) with a central frequency of 7 MHz, ~80% detection bandwidth, and a 40-mm focal distance (spherical radius). The array was mounted on a motorized *XYZ* translation stage (IAI Inc., Shizuoka Prefecture, Japan) and immersed in a custom-designed water tank with a polyethylene-sealed central aperture. Ultrasound gel and degassed water were used to ensure optimal acoustic coupling between the murine brain and the detector. Multispectral (multiwavelength) optical excitation was provided by an OPO laser (Spitlight EVO-II, Innolas GmbH, Krailling, Germany), operating at 100 Hz with an ~7-ns pulse duration. Laser light was delivered through a customized fiber bundle (active diameter: 5.85 mm; numerical aperture: 0.22; Lightguide GmbH, Meckenheim, Germany) inserted into the central cavity of the transducer array. For assessing the suitable wavelengths for OA excitation, the optimal excitation wavelength was screened for whole-brain vascular imaging. For imaging the entire murine cortex, the transducer was translated along three axes to locate nine brain imaging positions with a 2.5-mm step. In vivo OA tracking of magnetic dye-labeled blood cells under an external magnetic field was conducted in the flattened ear of anesthetized nude mice. On the imaging side, the ear surface was directly coupled with ultrasound gel for pulsed laser irradiation and OA signal detection. On the opposite side, a glass slide was placed against the ear surface, and a grade N42 neodymium permanent bar magnet (40 mm by 10 mm by 10 mm) was positioned 0.37 mm from the tissue and manually translated to enable magnetic manipulation. The generated OA signals were digitized at 40 megasamples per second using a custom data acquisition system (Falkenstein Mikrosysteme GmbH, Taufkirchen, Germany), synchronized via the Q-switch output of the laser.

### Localization and tracking

Acquired OA signals were structured as a 3D matrix comprising 493 temporal samples × 512 transducer elements × *N* frames. Initial signal preprocessing included a Butterworth bandpass filter (0.2 to 8 MHz) to suppress out-of-band noise, followed by SVD filtering to remove tissue clutter and motion artifacts, retaining eigenvectors of 31 to 1200, which corresponded to signals from fast-moving labeled cells (fig. S12). Baseline offset was removed in angular sectors (128 elements each), and 3D volumes (40 μm/pixel) were reconstructed and temporally summed to generate MC-OA images. A baseline offset correction was applied to each 128-element angular sector (~90°) of the transducer. Reconstructed volumetric frames (resolution of 40 μm/pixel) were temporally summed to generate MC-OA images. For depth visualization, voxels were color coded by their distance from the manually segmented tissue surface.

Superresolution LOT was performed by detecting local maxima post-SVD, correlating them with the system’s point spread function, and applying subvoxel fitting. Points with correlation coefficients >0.3 were retained and aggregated across frames. To quantify blood flow, localized cells were linked into trajectories using the Munkres algorithm. Instantaneous velocities were calculated on the basis of displacement across frames and compiled into a 3D cortical VM. Large-scale imaging was achieved by stepwise acquisition over nine positions. Reconstructed volumes were spatially registered using the MATLAB function imtranslate and combined via MIP. Localization and tracking of ICG-stained RBCs with DOLI were performed using a simple and extendable open-source framework TrackNTrace (*69*).

### Wavelength selection and multiplexed cell tracking

On the basis of preliminary multispectral tests, ICG- and DiR-labeled cells were separately injected into mice to identify wavelength-dependent detection characteristics. ICG-labeled cells could be reliably detected at two excitation wavelengths, whereas DiR-labeled cells were detectable at only one of these wavelengths. These results guided the selection of wavelength channels, which were subsequently implemented in the acquisition sequence. Using this tailored wavelength sequence, simultaneous injection of ICG- and DiR-labeled cells enabled multiplexed tracking and discrimination of their trajectories in vivo.

### DOLI imaging setup

The DOLI system was built on a conventional wide-field microscopy configuration (*35*). A 795-nm fiber-coupled continuous-wave laser (FC-795-5W, CNI Laser, China) served as the excitation source for in vivo imaging of ICG-labeled RBCs. Emitted FL was collected using a scan lens (LSM03-BB, Thorlabs, US) and a NIR-II coated camera lens (LM50HCSW, 50-mm focal length, Kowa, Japan) with at a fixed magnification of 1.39. A longpass filter (FELH0900, Thorlabs, US) was used to transmit emission signals above 900 nm. The filtered FL signal was then captured by a SWIR camera (WiDy SenS 640V-ST, New Imaging Technologies, France) at a frame rate of 69 Hz.

For imaging DiR-labeled RBCs, a 750-nm fiber-coupled continuous-wave laser (FC-750-5W, CNI Laser, China) was used as the excitation source. The output beam was passed through a bandpass filter (FBH750-10, Thorlabs, US) to irradiate the sample, and the resulting emission was filtered by a longpass filter (FELH0775, Thorlabs, US) before being recorded by a scientific complementary metal-oxide-semiconductor camera (pco.dimax S1, PCO AG, Germany) operating at 100 Hz.

### Magnetically induced ischemic stroke model

C57BL/6 mice were anesthetized and secured in a stereotaxic frame. An acute cranial window was created over the cerebral cortex using standard surgical procedures. Bright-field images were acquired using a SteREO Discovery.V20 stereomicroscope (Carl Zeiss Microscopy GmbH, Jena, Germany) equipped with a complementary metal-oxide-semiconductor camera (UI-1240LE-C-HQ, IDS Imaging Development Systems GmbH, Obersulm, Germany). These images were recorded as baseline measurements. Subsequently, one pole of a permanent magnet (49 mm by 17 mm by 5 mm) was positioned adjacent to the cranial window, while the opposite end was supported by a custom holder to prevent mechanical compression of the cortical surface and associated vessels. Magnetically labeled cells (150 μl, ~3 × 10^8^ cells/ml) were then intravenously injected via the tail vein. The magnet was maintained in a fixed position for 30 min, after which imaging was performed to assess changes in the brain. The ischemic lesion volume was quantified 24 hours poststroke. After euthanasia, brains were rapidly extracted and cut into 2-mm-thick coronal sections. Sections were incubated for 10 min in 4% TTC at 37°C. Following staining, sections were imaged, and infarcted tissue was identified as unstained (white) regions, clearly distinguishable from viable (red) tissue.

### Data analysis and statistics

Data processing and analysis were performed in MATLAB R2022a (MathWorks, US). Vessel quantification was conducted using AngioTool64 (version 0.6a), and plot profile analysis was carried out with ImageJ (National Institutes of Health, US). GraphPad Prism (version 8.1.0, GraphPad Software, US) was used for statistical analysis. Statistical significance was determined as follows: ns, not significant; *P < 0.05; **P < 0.01; ***P < 0.001; ****P < 0.0001. In the box-and-whisker plot, the central line represents the median (Q2), the dot indicates the mean, and the box spans the interquartile range (Q1 to Q3). Whiskers extend to the most extreme data points within the 1.5× interquartile range from the quartiles.
