# Supplementary material for: Multiplexed optoacoustic tracking and magnetic actuation of labeled blood cells in living mice
Source: Sci Adv. 2026 Jun 26;12(26):eaec8985. doi: 10.1126/sciadv.aec8985 (PMC13308621; doi:10.1126/sciadv.aec8985)
Supplement: Supplementary file 1 — Figs. S1 to S12 Table S1 Legends for movies S1 to S7 [file sciadv.aec8985_sm.pdf]

Science Advances
AAAS

Supplementary Materials for

Multiplexed optoacoustic tracking and magnetic actuation of labeled blood cells in living mice

Lin Tang *et al.*

Corresponding author: Daniel Razansky, daniel.razansky@uzh.ch; Xosé Luís Deán-Ben, xl.deanben@pharma.uzh.ch

Sci. Adv. **12**, eaec8985 (2026)
DOI: 10.1126/sciadv.aec8985

The PDF file includes:

Figs. S1 to S12
Table S1
Legends for movies S1 to S7

Other Supplementary Material for this manuscript includes the following:

Movies S1 to S7

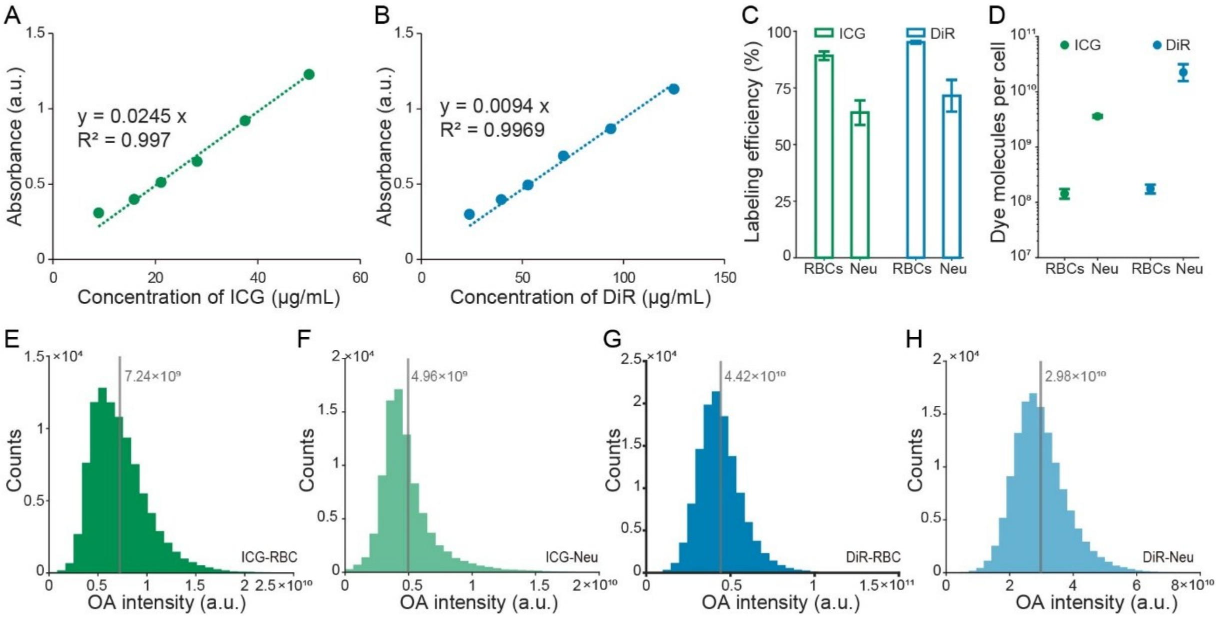

Fig. S1. Quantitative characterization of dye-labeled blood cells. (A) Standard calibration curve of absorbance at 780 nm as a function of ICG concentration. (B) Standard calibration curve of absorbance at 750 nm as a function of DiR concentration. (C) Labeling efficiency of ICG- and DiR-labeled RBCs and neutrophils (Neu). (D) Average number of dye molecules per collected cell, calculated based on absorption quantification and total cell counts. (E-H) OA signal intensity histograms for ICG-labeled RBCs (E), ICG-labeled Neu (F), DiR-labeled RBCs (G) and DiR-labeled Neu (H) measured from sparsely flowing cells in microtubing using the optoacoustic system.

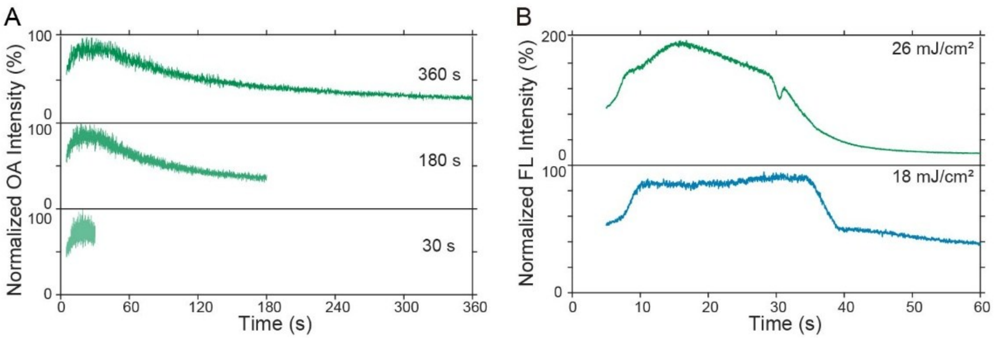

Fig. S2. Signal stability of ICG-labeled RBCs under pulsed laser irradiation. (A) Stability of OA signal. (B) Stability of FL signal.

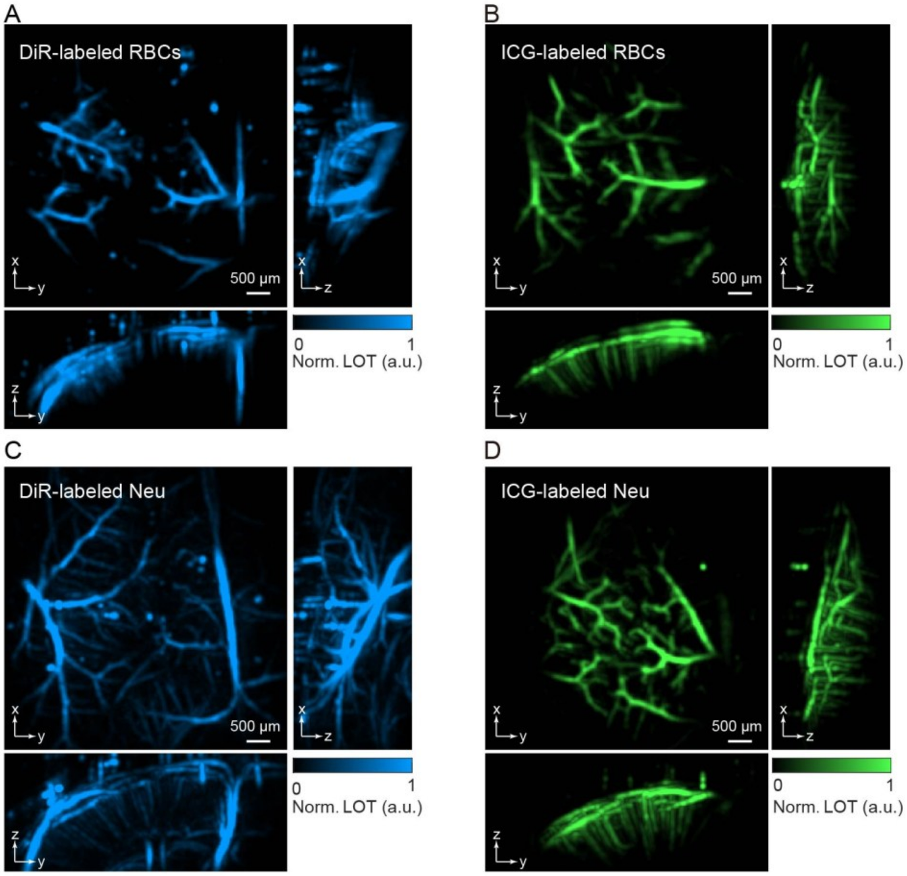

Fig. S3. OA images superimposing all localized cells from all wavelength channels (700–860 nm) during the acquisition period. (A) DiR-labeled RBCs**.** (B) ICG-labeled RBCs. (C) DiR-labeled Neu. (D) ICG-labeled Neu. Each panel shows maximum intensity projections (MIPs) views in the xy, yz, and zx planes. Note: Panels A (DiR-labeled RBCs) and B (ICG-labeled RBCs) show vessel structures similar to those in Fig. 1F and H, respectively, as they correspond to the same field of view. The differences arise from distinct spectral channels: the former represents the combined signal across multiple wavelengths (700–860 nm), whereas the latter reflects signals detected at a single wavelength.

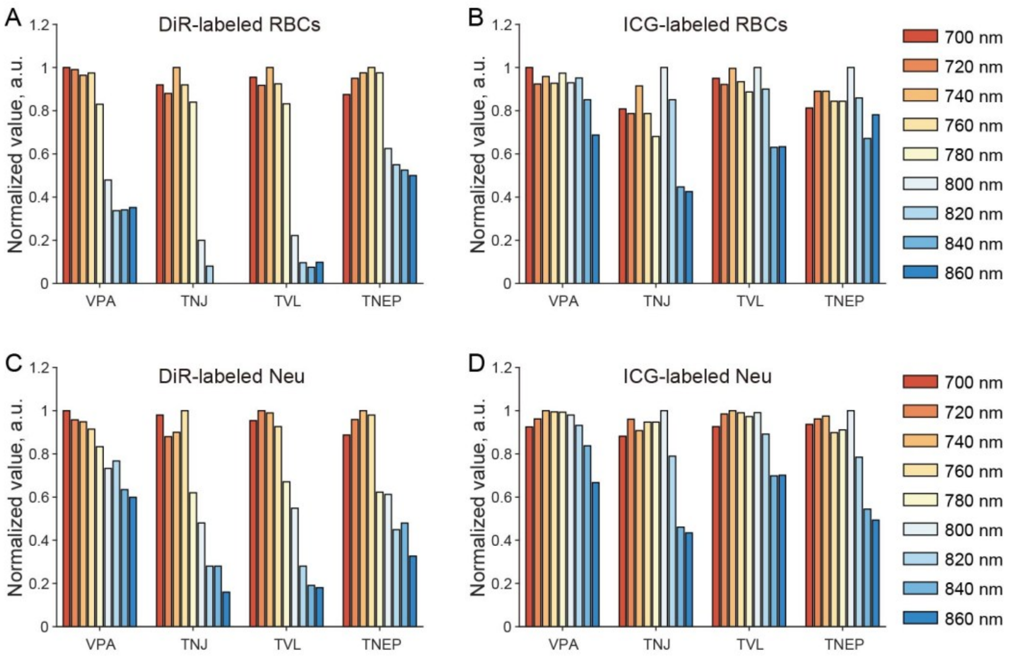

Fig. S4. Vessel quantification from OA images represented as xy-plane MIPs, superimposing all localized cells at each excitation wavelength (700-860 nm) over the acquisition period. (A) DiR-labeled RBCs. (B) ICG-labeled RBCs. (C) DiR-labeled Neu. (D) ICG-labeled Neu. VPA, vessel percentage area; TNJ, total number of junctions; TVL, total vessel length; TNEP, total number of endpoints.

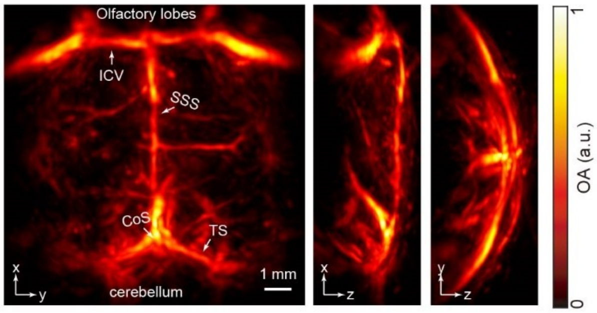

Fig. S5. Whole-brain optoacoustic tomography image reconstructed by stitching raw data from nine acquisition positions. Anatomical structures labeled: ICV, inferior cerebral veins; SSS, superior sagittal sinus; CoS, confluence of sinuses; TS, transverse sinuses.

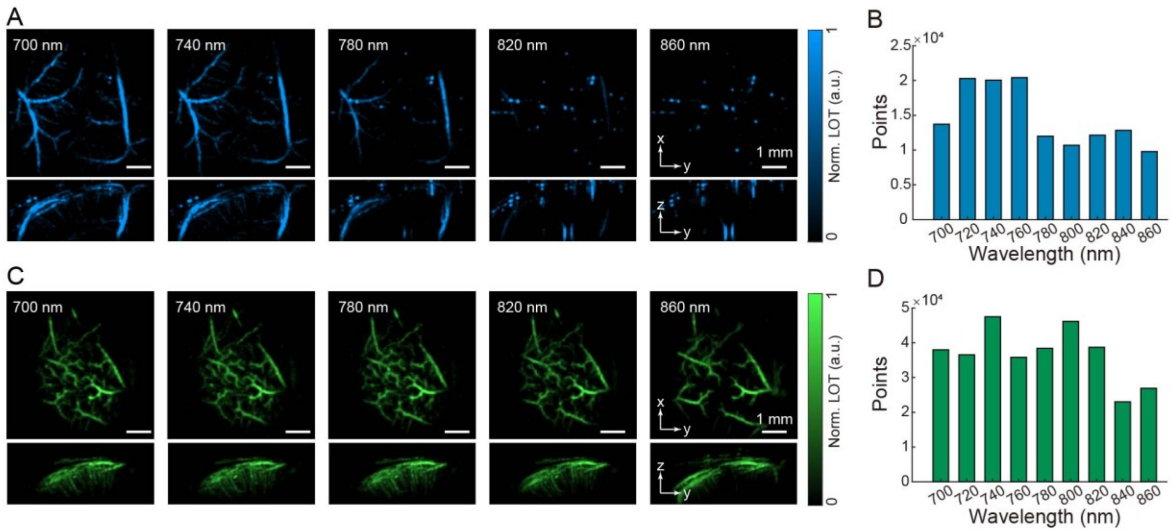

Fig. S6. *In vivo* multispectral localization optoacoustic performance of dye-labeled neutrophils. (A) MIPs of detected DiR-labeled neutrophils in mouse brain vessels, extracted at individual excitation wavelengths (700, 740, 780, 820, and 860 nm) from multispectral image sequences acquired with repeated wavelength cycling between 700 and 860 nm during the imaging session. (B) Total number of DiR-labeled neutrophils detected at each wavelength. (C) MIPs of ICG-labeled neutrophils extracted at the same set of excitation wavelengths from multispectral image sequences acquired under identical conditions. (D) Total number of ICG-labeled neutrophils detected at each wavelength. Note: Panels A (DiR-labeled Neu) and C (ICG-labeled Neu) show vessel structures similar to those in Fig. S3C and D, respectively, as they correspond to the same field of view. The differences arise from distinct spectral channels: the former reflects signals detected at a single wavelength, while the latter represents the combined signal across multiple wavelengths (700–860 nm).

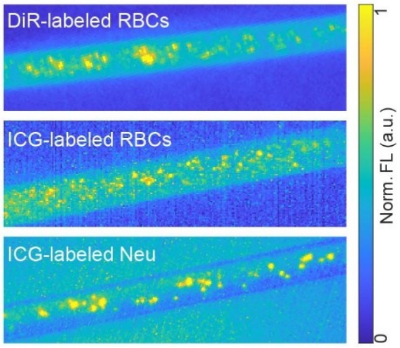

Fig. S7. Fluorescence images of dispersed dye-labeled cells detected within capillary tubing.

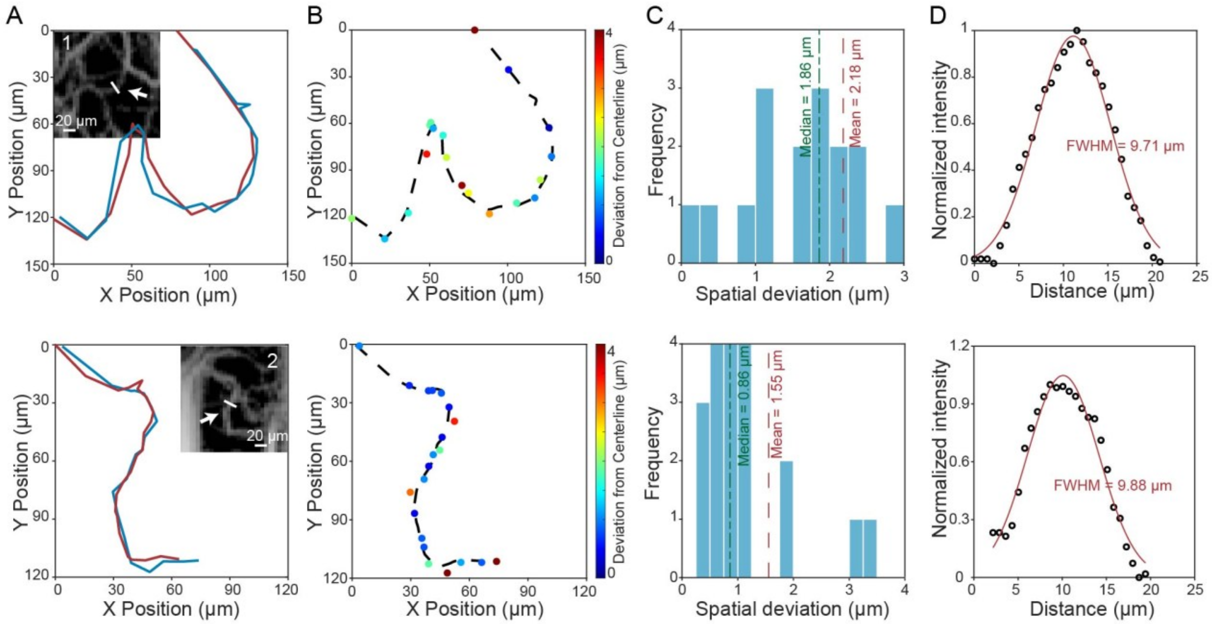

Fig. S8. Quantitative assessment of capillary diameters and spatiotemporal RBC trajectories. (A) Accumulated spatiotemporal trajectories of ICG-labeled RBCs traversing the selected capillaries during the entire observation period. Inset: representative zoomed-in regions highlighting capillary 1 and capillary 2, as indicated by white arrows. (B) Maps of lateral spatial deviations along the extracted vessel centerlines. The color scale denotes the positional offset of individual RBCs relative to the computed capillary centerline. (C) Statistical distribution of the measured lateral spatial deviations. (D) Representative cross-sectional intensity profiles (black circles) extracted along the white lines shown in the inset of (A). Solid curves correspond to Gaussian fits used for vessel diameter estimation. The Full Width at Half Maximum (FWHM) values are indicated for each capillary.

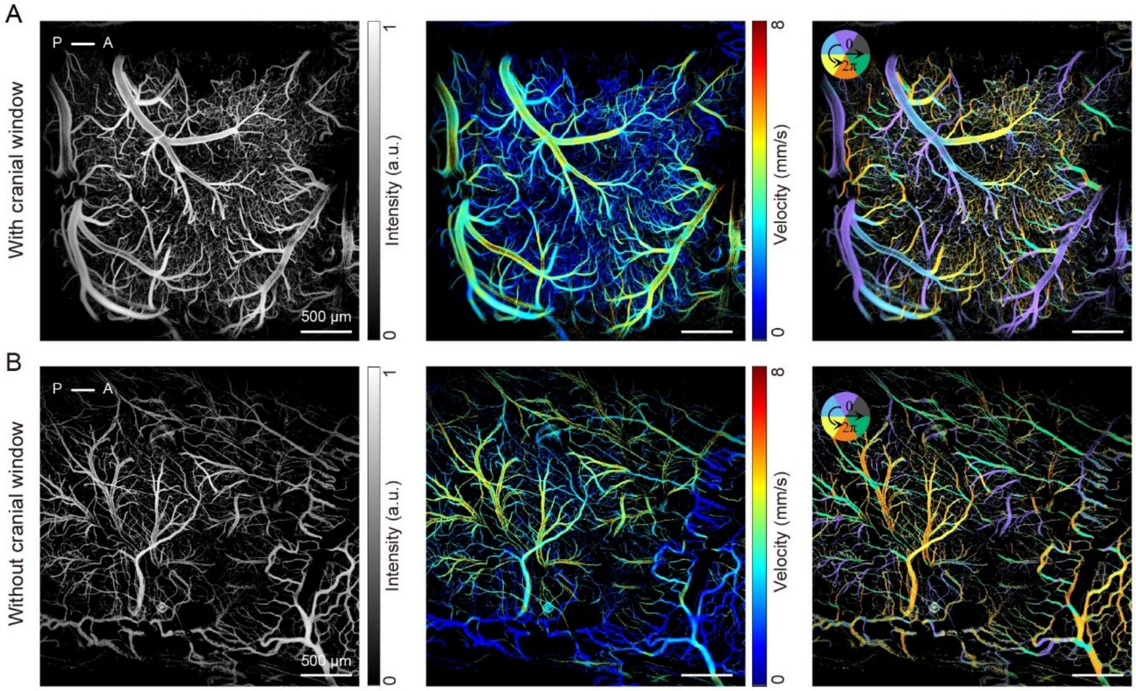

Fig. S9. Fluorescence localization imaging of DiR-labeled RBCs in the NIR-I window. (A) Localization-reconstructed vascular structure, color-encoded flow velocity and direction maps of the mouse brain through a cranial window, respectively. (B) Localization-reconstructed vascular structure, color-encoded flow velocity and direction maps of the mouse brain through the intact skull with scalp removed, respectively.

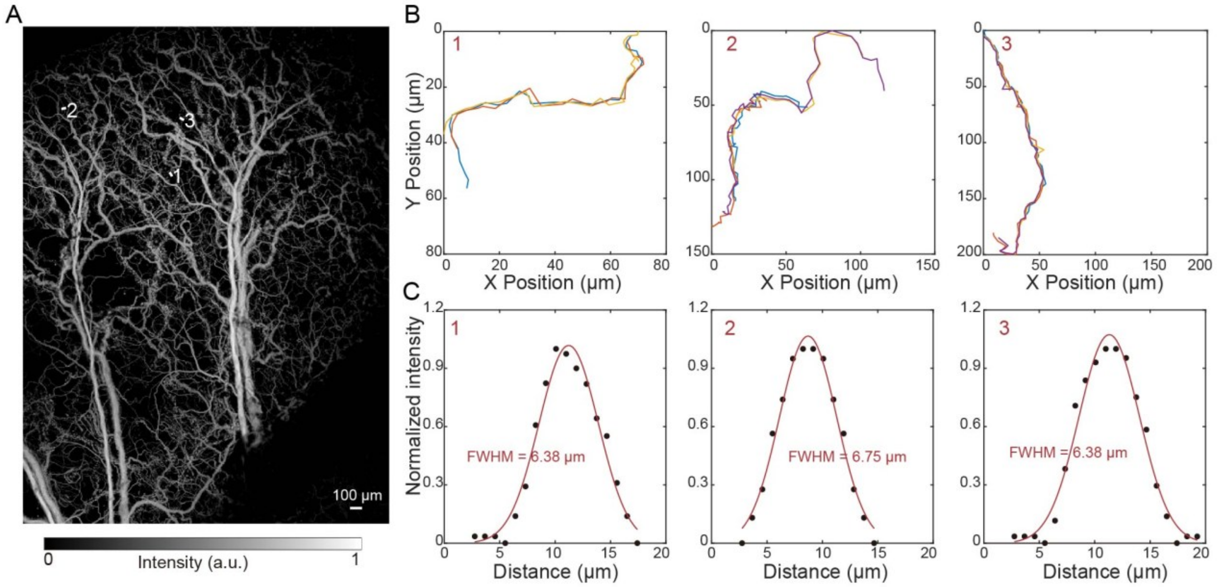

Fig. S10. ***In vivo*** **assessment of capillary transit and deformability of Fe_3O_4@PDA-modified RBCs.** (A) Fluorescence localization imaging of mouse ear microvasculature following intravenous injection of Fe_3O_4@PDA-modified RBCs. Three representative capillaries were selected for quantitative analysis (labeled by 1, 2 and 3). (B) Cumulative spatiotemporal trajectories of modified RBCs traversing the selected capillaries over the imaging period, demonstrating successful capillary passage without persistent obstruction. (C) Representative cross-sectional intensity profiles extracted along the white lines shown in (A). Solid curves represent Gaussian fits used to estimate capillary diameters.

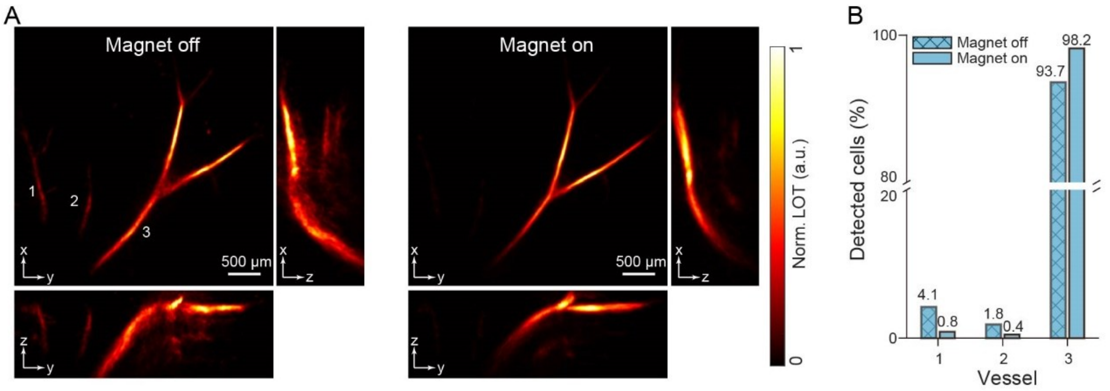

Fig. S11. Magnetic actuation of DiR-labeled RBCs *in vivo*. (A) LOT images of the same vascular region with and without magnetic field. (B) Distribution of detected cells across vessels 1–3 (as indicated in panel A) under magnet off and magnet on conditions. Percentages were calculated relative to the total number of detected cells in the field of view. Magnet off: 28196 cells; magnet on: 21451 cells.

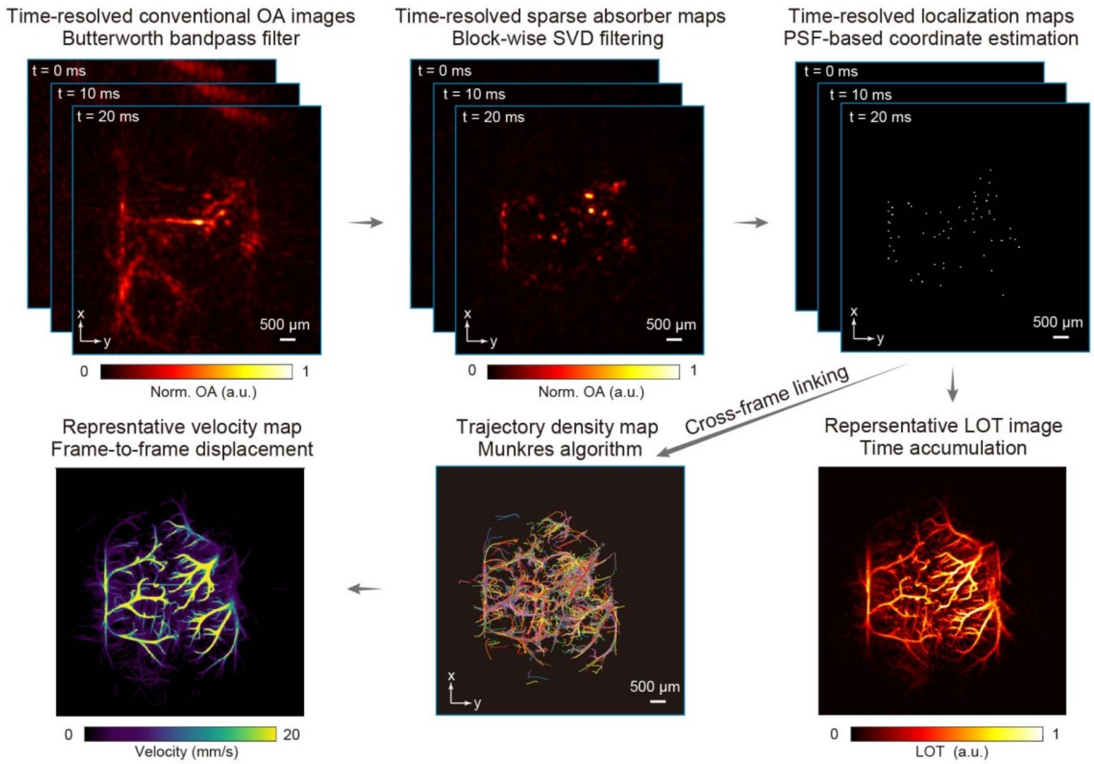

Fig. S12. Schematic illustration of the data processing pipeline for LOT. The workflow includes bandpass filtering, SVD-based background suppression, sub-voxel position estimation, trajectory linking, and velocity mapping. Note: The LOT and velocity maps shown here correspond to a subregion of the whole-brain imaging presented in Fig. 2 and are reused here to illustrate the data processing workflow.

Table S1. Summary of imaging performance parameters of the systems.

Metric	Depth	LOT	DOLI
Penetration depth	-	3.2 mm	-
Lateral resolution	0 mm	24 μm	9.57 μm
	1 mm	24 μm	
	2 mm	24 μm	
	3 mm	40 μm	
Axial resolution	0 mm	32 μm	-
	1 mm	32 μm	
	2 mm	32 μm	
	3 mm	40 μm	
Temporal resolution	-	4 s	2 s
Imaging speed	-	100 Hz	69 Hz
Signal sensitivity (SNR)	-	19.61 ± 8.17	4.15 ± 1.21

Data are expressed as mean ± SD.

SUPPLEMENTARY MOVIES

Supplementary Movie 1. *In vivo* visualization of the dynamic flow of individual ICG-labeled RBCs. The red signal originates from intrinsic hemoglobin contrast, while the green signal represents ICG-labeled RBCs following SVD filtering.

Supplementary Movie 2. Rotating 3D-views of MC-OA image of the mouse brain vasculature and the corresponding blood flow velocity map.

Supplementary Movie 3. Fluorescence imaging of flowing DiR-labeled RBCs within capillary tubing.

Supplementary Movie 4. Fluorescence imaging of flowing ICG-labeled RBCs within capillary tubing.

Supplementary Movie 5. Fluorescence imaging of flowing ICG-labeled neutrophils within capillary tubing.

Supplementary Movie 6. *In vivo* localization fluorescence imaging of mouse cerebral vasculature at 1 min and 40 min after injection of ICG-labeled RBCs.

Supplementary Movie 7. *In vivo* optoacoustic imaging of ICG-labeled RBCs under magnetic actuation.
